# Comparison of the efficacy of spinal cord stimulation and dorsal root ganglion stimulation in the treatment of painful diabetic peripheral neuropathy: a prospective, cohort-controlled study

**DOI:** 10.3389/fneur.2024.1366796

**Published:** 2024-04-10

**Authors:** Yu-Fei Han, Xi Cong

**Affiliations:** Department of Neurosurgery, Shengjing Hospital of China Medical University, Shenyang, Liaoning, China

**Keywords:** dorsal root ganglion stimulation, neuropathic pain, diabetic peripheral neuropathy, spinal cord stimulation, PDPN

## Abstract

**Objective:**

The aim of this study was to compare the clinical outcomes of spinal cord stimulation (SCS) and dorsal root ganglion stimulation (DRG-S) in the treatment of painful diabetic peripheral neuropathy (PDPN).

**Methods:**

In this prospective cohort study, 55 patients received dorsal column spinal cord stimulation (SCS group) and 51 patients received dorsal root spinal cord stimulation (DRG-S group). The primary outcome was a Numerical Rating Scale (NRS) remission rate of ≥50%, and secondary outcomes included the effects of SCS and DRG-S on quality of life scores (EQ-5D-3L), nerve conduction velocity, and HbA1c, respectively.

**Results:**

The percentage of NRS remission rate ≥ 50% at 6 months was 80.43 vs. 79.55%, OR (95% CI): 1.06 (0.38–2.97) in the SCS and DRG-S groups, respectively, and the percentage of VAS remission rate ≥ 50% at 12 months was 79.07 vs. 80.95%, OR (95% CI): 0.89 (0.31–2.58). Compared with baseline, there were significant improvements in EQ-5D and EQ-VAS at 6 and 12 months (*p* < 0.05), but there was no difference in improvement between the SCS and DRG-S groups (*p* > 0.05). Nerve conduction velocities of the common peroneal, peroneal, superficial peroneal, and tibial nerves were significantly improved at 6 and 12 months compared with the preoperative period in both the SCS and PND groups (*p* < 0.05). However, at 6 and 12 months, there was no difference in HbA1c between the two groups (*p* > 0.05).

**Conclusion:**

Both SCS and DRG-S significantly improved pain, quality of life, and lower extremity nerve conduction velocity in patients with PDPN, and there was no difference between the two treatments at 12 months.

## Introduction

As the world’s population ages, the latest data for 2021 estimate that 536.6 million people worldwide will have diabetes (a prevalence of 10.5 percent) (1). Painful diabetic peripheral neuropathy (PDPN) occurs in 25 percent of people with diabetes and is a progressive neurological disorder with neuropathic pain symptoms; PDPN manifests as pain and other sensory dysfunctions, including numbness, burning, or tingling. It often leads to insomnia, poor quality of life, mood disorders, and even falls, with an increased risk of foot ulcers and lower limb amputation, with far-reaching health-related quality of life implications and potentially life-threatening consequences (2). International guidelines recommend amitriptyline, duloxetine, pregabalin, or gabapentin as first-line symptomatic analgesics for patients with DPNP. According to a Cochrane review, the best outcome of any single-agent treatment is 50% pain relief in less than half of patients, with medication restrictions and serious adverse effects (3). This makes the treatment of DPNP difficult.

Over the years, neuromodulation has achieved remarkable results in the treatment of chronic pain. According to the ‘gate control theory’ proposed by Melzack and Wall (4), which proposed that epidural electrodes placed on the dorsal side of the spinal cord could disrupt the transmission of nociceptive signals by reversing the stimulation of inhibitory interneurons through Aβ fibres in the spinal cord, thereby attenuating the transmission of nociceptive signals from the spinal cord to the brain. Randomised controlled trials have shown that SCS can result in pain relief of more than 50% on the Numeric Rating Scale (NRS) in approximately 70% of patients with PDPN and can significantly improve quality of life (EQOL-5D) (5–10). Dorsal root ganglion stimulation (DRG-S) is a novel neuromodulation therapy that targets the dorsal root ganglion (DRG) before the afferent spinal sensory neurons. This is very different from SCS, which targets dorsal column fibres. DRG-S is therefore also a valuable neuromodulation intervention for chronic neuropathic pain. In a number of mixed aetiology cohorts, DRG-S has been shown to provide relief of neurogenic pain in complex regional pain syndrome (CRPS), complex regional pain syndrome of the lower extremities, and chronic post-operative pain within 12 months (11–15). Recent studies have shown that the mechanism of pain relief by DRG-S is not dependent on GABA release and it is hypothesised that it may be due to the induction of conduction block via the C-type T junction located in the DRG itself, which acts as a low-pass filter for the conduction of action potentials (nociceptive signals) from the periphery to the spinal cord (16, 17).

Unfortunately, most previous clinical trials of SCS and DRG-S have focused only on the effect of implanted electrodes on the level of pain relief in patients with PDPN, neglecting improvements in quality of life, lower limb nerve conduction, and HbA1c. Therefore, we prospectively conducted this study of SCS and DRG-S for the treatment of PDPN, and our primary endpoint was to compare the proportion of NRS remission ≥50% at 6 and 12 months postoperatively between the two groups, and the secondary endpoints were to analyse the quality of life scores (EQOL-5D-3L), HbA1c, and nerve conduction velocity in the peripheral nerves of the lower limbs. This clinical trial comparing SCS and DRG-S for the treatment of PDPN provides the latest research on the use of different neuromodulation techniques in the treatment of lower limb pathologic pain and provides clinicians with more clinical decision support when individualising treatment for patients with PDPN.

## Materials and methods

### Research design

This is a prospective, cohort-controlled study. The study was conducted from January 2020 to January 2023 at the Neurosurgery Outpatient Clinic of Shengjing Hospital, China Medical University. The study was approved by the medical ethics committee of the hospital (2019PS869J), and the Declaration of Helsinki was adhered to in all procedures. All patients signed an informed consent form before participating in the study. To reduce data bias, two physicians used a blinding method to collect patient information and repeated the measurements multiple times to reduce random errors.

Patients’ pain levels were assessed preoperatively and at 6 and 12 months postoperatively using the Numerical Rating Scale (NRS), the most widely used scale for the assessment of chronic pain. The European 5-Dimensional Quality of Survival Scale (EQ-5D-3L) is the most widely used scale for measuring health-related quality of life and consists of the EQ-5D descriptive system (five dimensions of mobility, self-care, activities of daily living, pain, anxiety, and depression, each with three levels) and a visual scale (EQ-VAS). The EQ-VAS is a visual analogue scoring tool used to assess a patient’s overall subjective perception of their health. 0 represents the worst health and 100 represents the best health. Lower limb nerve conduction velocity is measured using electrodes placed at fixed locations on the patient’s lower limbs to detect bioelectrical signals in the muscles at rest or during contraction. Lower limb nerve conduction velocity is measured in metres per second (m/s), with slower velocities indicating more severe lower limb neuropathy.

### Participants

Inclusion criteria were (1) age 18–80 years. (2) Diagnosis of painful diabetic peripheral neuropathy and stable glycaemic control with glycaemic haemoglobin (HbA1c) below 10% in the previous 3 months. (3) Unsatisfactory pain relief with conventional medications with an NRS score ≥ 5 (NRS: 0 means ‘no pain’ and 10 means ‘worst pain imaginable’). (4) Consent to participate in the study and actively participate in the postoperative follow-up. The exclusion criteria were: (1) Diagnosis of other painful peripheral neuropathies based on clinical history and review of medical records. (2) Contraindications to surgery or inability to tolerate surgical treatment, such as cardiopulmonary dysfunction. (3) Pregnancy, lactation and severe systemic disease.

### Surgical procedures

#### SCS group

A paddle-like SCS surgical lead (SPECIFY 5-6-5, Medtronic, Inc.) was inserted into the epidural space through an intervertebral midline approach while the patient was under local anaesthesia and in the prone position. The Specify 5-6-5 lead was placed over the spinal cord segment receiving the dorsal root fibres (T10-T12) corresponding to the area of pain. The stimulation protocol was individualised for each patient in order to provide maximum relief from neurogenic pain. One week after the stimulation trial, the previous incision was reopened and the implanted leads were connected to the implantable pulse generator (IPG) using a lead connector.

#### DRG-S group

Under general anaesthesia, two wires (SPECIFY 2 × 8, Medtronic, Inc.) were advanced into the epidural space under fluoroscopic guidance until they entered the intervertebral foramina near the lumbar ganglion at L4-L5 bilaterally. As part of intraoperative device programming, the appropriate location of the lead was determined by overlapping areas of paresthesia and pain. If overlap of pain and paresthesia was not achieved, the lead was repositioned and reprogrammed under fluoroscopy, and the IPG was implanted 1 week after the trial.

#### Device programming

Pre-programming of the devices was performed by a clinical device technician employed by the device manufacturer according to an established protocol. Individual reprogramming (40–60 Hz, 180–240 μs, 0.5–2.0 V) was performed in both groups of patients using a programming controller (Medtronic, model 97745) under remote control of the technician.

### Statistical methods

Based on the results of a previous study in which the proportion of SCS relieving NRS ≥50% was 60% (5–7), with a two-sided test level of *α* = 0.05 and a power of 1−*β* = 0.8, a minimum of 90 participants would be required to determine the superiority or inferiority of the DRG-S, taking into account a 20% loss to follow-up rate, as calculated by SPSS.

Data were analysed using SPSS 22.0 software and plotted using GraphPad Prism. Continuous variables are expressed as mean (standard deviation). Categorical variables are expressed as percentages. Changes in between- and within-group variables were compared between the SCS and DRG-S groups at pre-treatment, 6 and 12 months post-treatment using paired-samples *t*-tests. Differences of *p* < 0.05 were statistically significant. Differences in primary endpoints were compared between groups using paired t-test analyses based on the minimum clinically important difference (MCID).

## Results

### Follow-up and baseline characteristics

**Figure 1:**
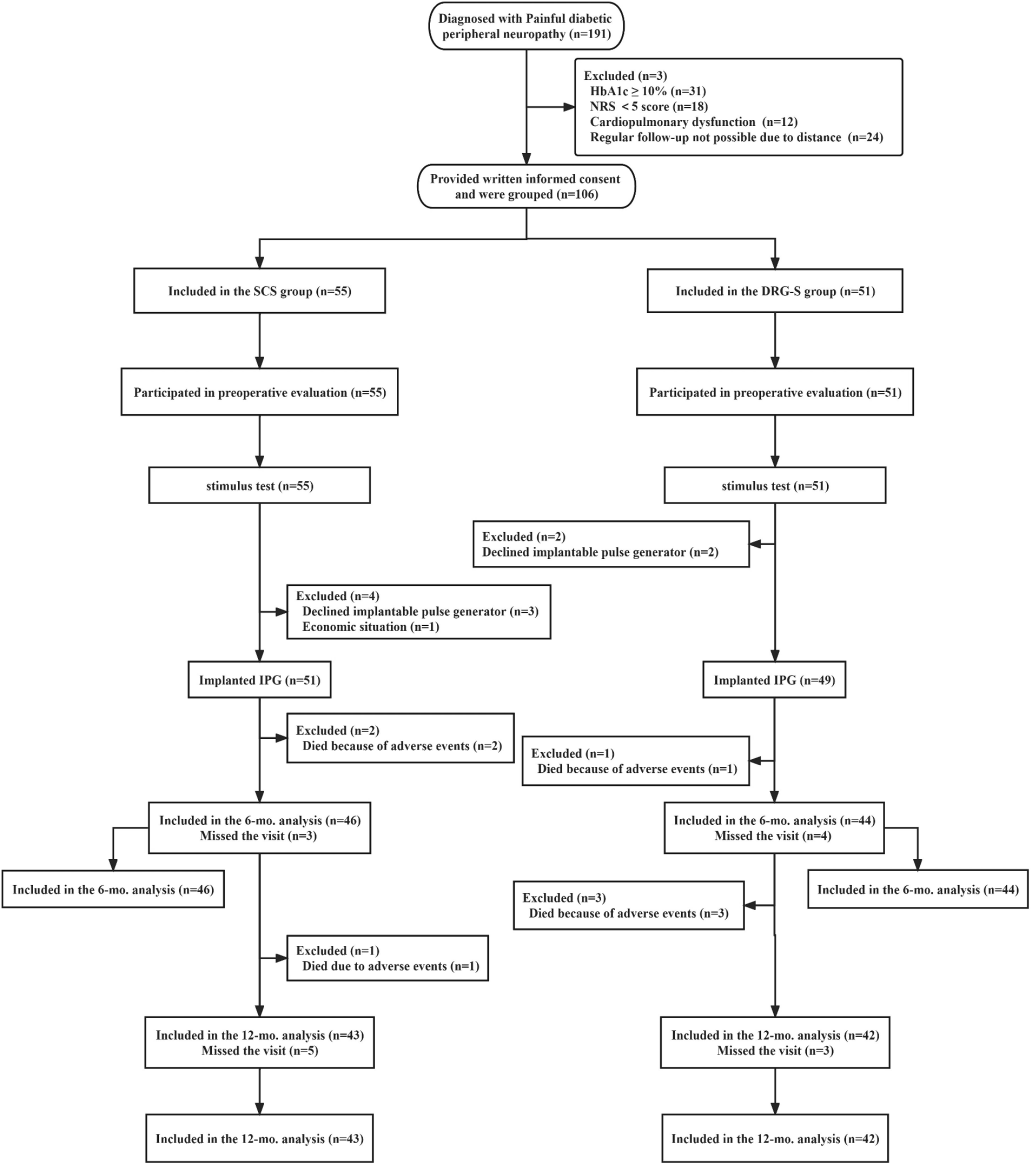
Flowchart of the study process. ^*^In this study, 46 people in the SCS group completed the 6-month follow-up and 43 people completed the 12-month follow-up; 44 people in the DRG group completed the 6-month follow-up and 42 people completed the 12-month follow-up.

Of the 191 screened patients, 106 with PDPN who met the inclusion criteria participated in this study: SCS group (*n* = 55), DRG-S group (*n* = 51). After the final stimulation trial, the SCS group (*n* = 51) and the DRG-S group (*n* = 49) underwent permanent implantation of IPGs (Figure 1). The baseline characteristics and PDPN-related medical history of patients in the 6- and 12-month follow-up groups, including age, sex, body mass index (BMI), duration of diabetes, duration of pain symptoms, glycated haemoglobin (HbA1c), type of diabetes, NRS score, and quality of life score (EQOL-5D-3L), were equipotent and comparable at *p* > 0.05 (Table 1).

**Table 1:** Patient demographics and baseline characteristics.

	Participation in 6-month follow-up			Participation in 12-month follow-up		
	SCS (*n* = 46)	DRG-S (*n* = 44)	*p* value	SCS (*n* = 43)	DRG-S (*n* = 42)	*p* value
Age (years), mean (SD)	65.35(11.38)	66.23 (10.01)	0.527	65.89 (11.55)	66.53 (10.48)	0.495
Men, *n* (%)	29 (63.04)	29 (65.91)	-	28 (65.12)	28 (66.67)	-
BMI (kg/m^2^), mean (SD)	24.27 (1.45)	23.82 (1.77)	0.486	24.34 (1.32)	24.03 (1.27)	0.548
Duration of diabetes (years), mean (SD)	16.16 (10.09)	18.84 (13.09)	0.127	16.23 (10.01)	18.05 (12.85)	0.408
Duration of pain (months), mean (SD)	4.52 (2.45)	4.03 (2.13)	0.513	4.48 (2.34)	4.07 (2.17)	0.486
HbA1c (%), mean (SD)	8.19 (0.64)	8.42(0.62)	0.223	8.23(0.69)	8.51 (057)	0.314
Types of diabetes						
Type 1, *n* (%)	8 (19.39)	6 (13.64)		6 (13.95)	5 (11.90)	
Type 2, *n* (%)	38 (80.61)	38(86.36)		37 (86.05)	37 (88.10)	
Opioid, *n* (%)	44 (95.65)	41 (93.18)		42 (97.67)	41 (97.62)	
NRS (score), mean (SD)	8.30 (0.98)	8.09 (1.29)	0.399	8.29 (1.00)	8.12 (1.27)	0.505
EQOL-5D-3L						
EQ-5D (score), mean (SD)	0.31 (0.13)	0.30 (0.11)	0.455	0.32 (0.13)	0.30 (0.11)	0.470
EQ-VAS (score), mean (SD)	57.59 (7.80)	56.09 (9.82)	0.438	57.50 (7.97)	56.36 (9.97)	0.569
Nerve conduction velocity						
Common peroneal nerve, m/s	34.44 (4.02)	36.51 (3.82)	0.108	34.62 (4.02)	36.25 (3.71)	0.055
Peroneal nerve, m/s	38.83 (3.44)	38.34 (1.80)	0.396	38.83 (3.52)	38.32 (1.75)	0.392
Superficial peroneal nerve, m/s	37.93 (4.85)	38.36 (2.97)	0.654	37.76 (4.86)	38.43 (3.02)	0.494
Tibial nerve, m/s	35.75 (3.37)	36.40 (3.67)	0.423	35.86 (3.39)	36.36 (3.74)	0.543

ABI, Ankle brachial index; BMI, Body mass index; HbA1c, Glycated haemoglobin; SD, Standard deviation; and NRS, Numerical rating scale.

### Numerical rating scale

**Figure 2:**
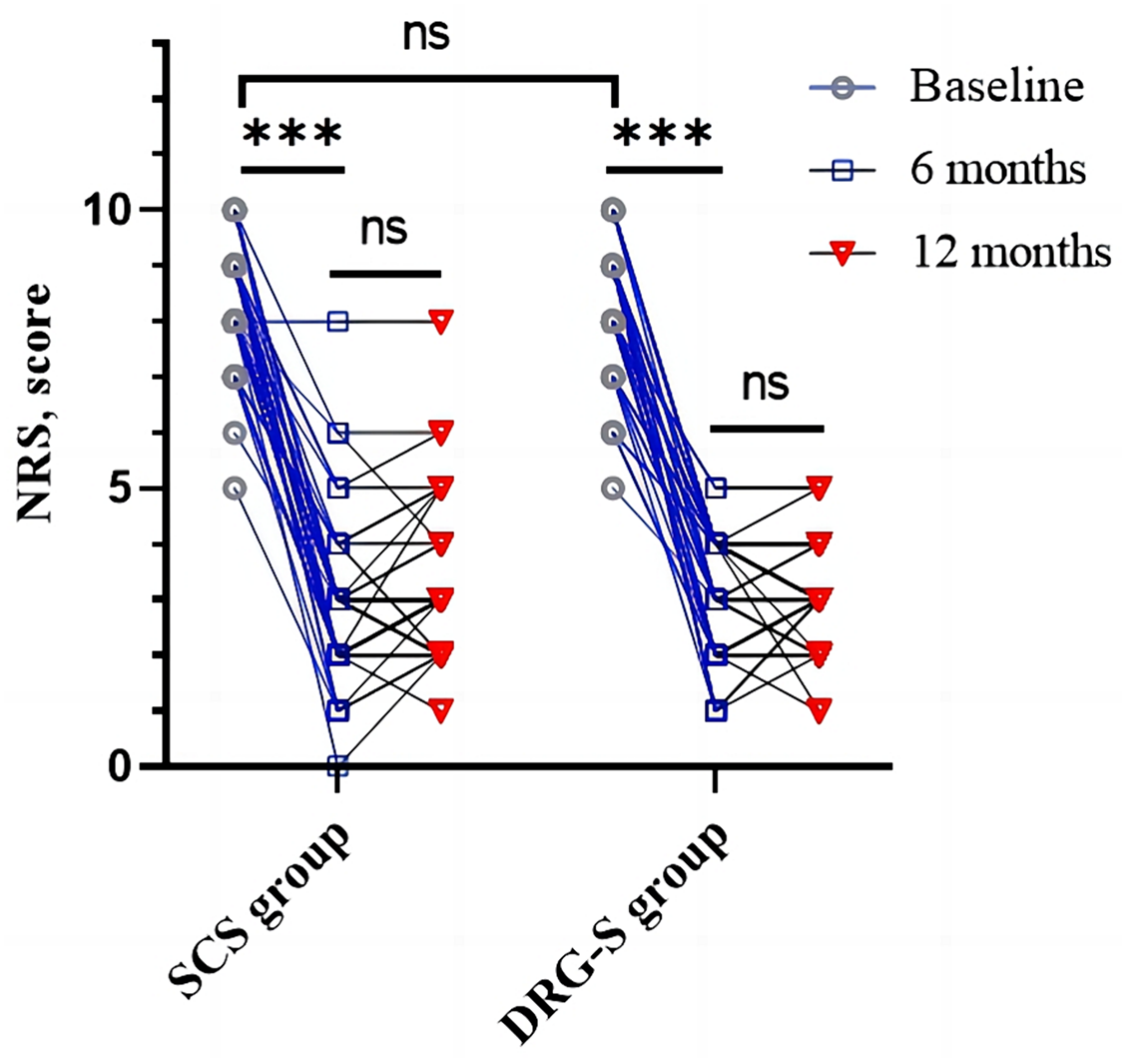
Changes in NRS in the SCS and DRG-S groups at baseline, 6 and 12 months. ^*^p < 0.05; ^**^p < 0.01; ^***^p < 0.001; NS, *p* > 0.05.

Regardless of whether the MCID for consideration of the NRS was 2 or the recently recommended 0.9–2.7 (18–20). At 6-month follow-up, there was a reduction of 5.32 (1.74) and 4.93 (1.65) in the SCS group of 2.98 (1.49) and the DRG-S group of 3.16 (1.10) compared with baseline, respectively, *p* < 0.001, however there was no difference in the degree of remission between groups, *p* = 0.325; at 12-month follow-up, there was a reduction of 5.05 (1.78) and 5.10 (1.48) in the SCS group 3.24 (1.41) and 3.02 (0.92) in the DRG-S group, respectively, *p* < 0.001, compared to baseline, and again there was no difference in the degree of remission between groups, *p* = 0.890 (Figure 2; Table 2).

**Table 2:** Changes in pain and quality of life at different time points in SCS and DRG-S groups.

Meae	It time point	SCS group		DRG-S group		*p* value
		Mean	SD	Mean	SD	
NRS system	6-month follow-up	2.98	1.49	3.16	1.10	0.508
	12-month follow-up	3.24	1.41	3.02	0.92	0.352
	Change from baseline at 6-month	5.32	1.74	4.93	1.65	0.325
	Change from baseline at 12-month	5.05	1.78	5.10	1.48	0.890
	Changes at 12-month compared to 6-month	0.29	1.11	0.10	1.08	0.132
EQ-5D system	6-month follow-up	0.58	0.10	0.61	0.10	0.116
	12-month follow-up	0.61	0.07	0.59	0.10	0.404
	Change from baseline at 6-month	0.27	0.14	0.32	0.15	0.077
	Change from baseline at 12-month	0.28	0.16	0.27	0.17	0.724
	Changes at 12-month compared to 6-month	0.03	0.12	0.04	0.15	0.180
EQ-VAS system	6-month follow-up	75.61	4.25	71.91	8.87	0.114
	12-month follow-up	74.88	6.50	70.36	6.19	0.086
	Change from baseline at 6-month	18.02	6.49	15.82	13.84	0.373
	Change from baseline at 12-month	17.38	8.70	14.00	12.75	0.206
	Changes at 12-month compared to 6-month	−0.64	5.92	−1.31	6.57	0.670

More importantly, we observed *a* ≥ 50% reduction in NRS at 6 months in 37 (80.43%) of SCS group 46 and 35 (79.55%) of DRG-S group 44, OR (95% CI): 1.06 (0.38–2.97) for both groups; and at 12 months in 34 (79. 07%) of SCS group 43 and 34 (80.95%) of DRG-S group 42 (Table 3), the NRS was reduced by ≥50%, OR (95% CI): 0.89 (0.31–2.58), which was not statistically different.

**Table 3:** Comparison of primary endpoints in the SCS and DRG-S groups.

		SCS group	DRG-S group	OR (95%CI)
6-month follow-up	NRS ≥ 50%, *n* (%)	37 (80.43%)	35 (79.55%)	1.06 (0.38–2.97)
	NRS<50%, *n* (%)	9 (19.57%)	9 (20.45%)	-
12-month follow-up	NRS ≥ 50%, *n* (%)	34 (79.07%)	34 (80.95%)	0.89 (0.31–2.58)
	NRS < 50%, *n* (%)	9 (20.93%)	8 (19.05%)	-

NRS, Numerical rating scale; OR, Odds ratio.

### Quality of life score (EQOL-5D-3L)

**Figure 3:**
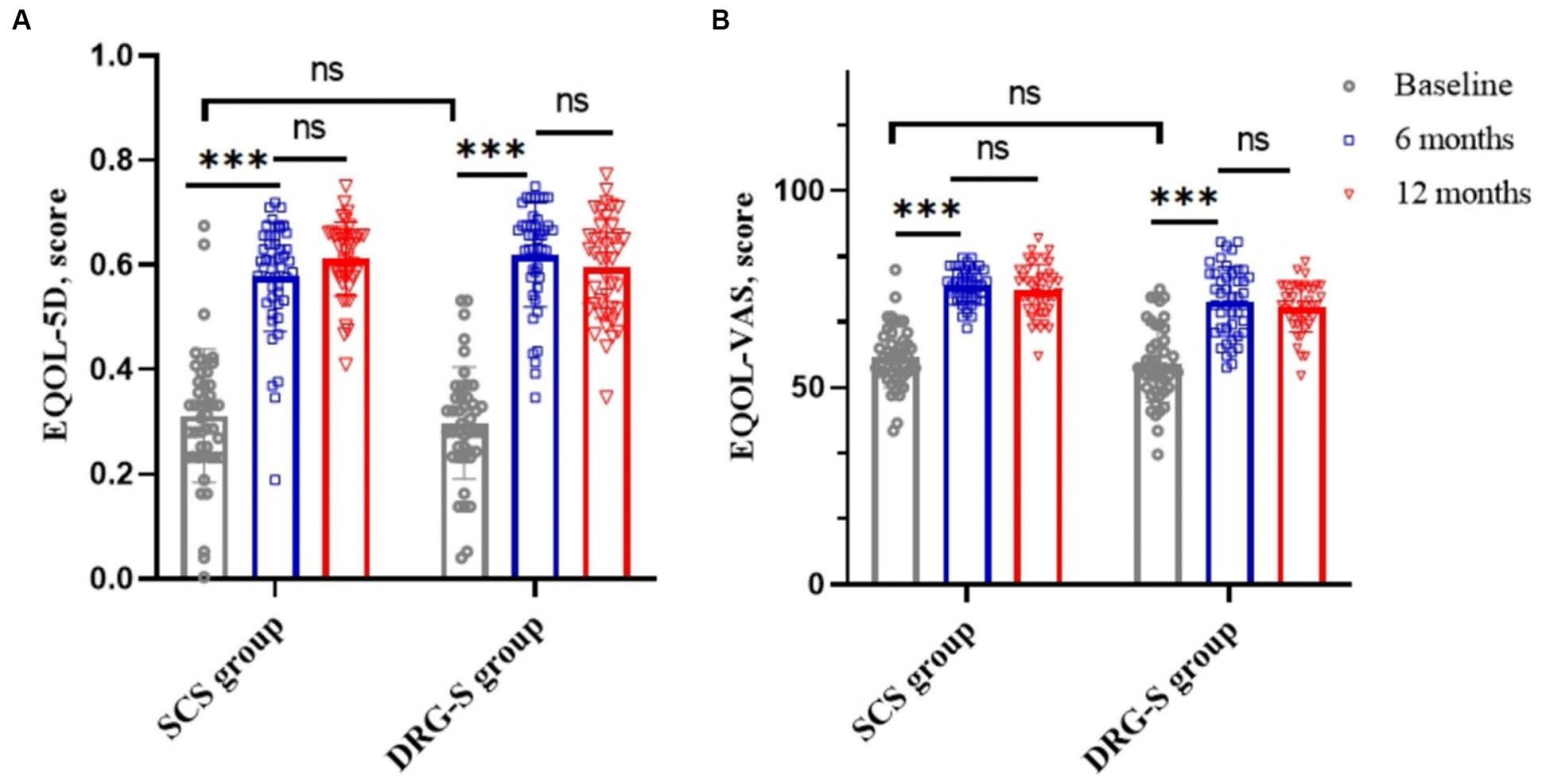
SCS and DRG-S group changes in EQ-5D and EQ-VAS at baseline, 6 and 12 months. ^*^p < 0.05; ^**^p < 0.01; ^***^p < 0.001; NS, *p* > 0.05. **(A)** Changes in EQ-5D at baseline, 6 and 12 months in the SCS and DRG-S groups; **(B)** Changes in EQ-VAS at baseline, 6 and 12 months in the SCS and DRG-S groups.

The MCID based on the EQOL-5D was 0.074 (21), which was a significant improvement (*p* < 0.001) compared to baseline at 6 months in the SCS group 0.58 (0.10) and in the DRG-S group 0.61 (0.10) and at 12 months in the SCS group 0.61 (0.07) and in the DRG-S group 0.59 (0.10). There was no significant difference between the two groups in terms of improvement at 6 and 12 months (Figure 3A; Table 2). Similarly, EQOL-VAS improved in both groups at 6 and 12 months postoperatively (Figure 3B), and there was no difference in improvement between the two groups (Table 2).

### Nerve conduction velocity

**Figure 4:**
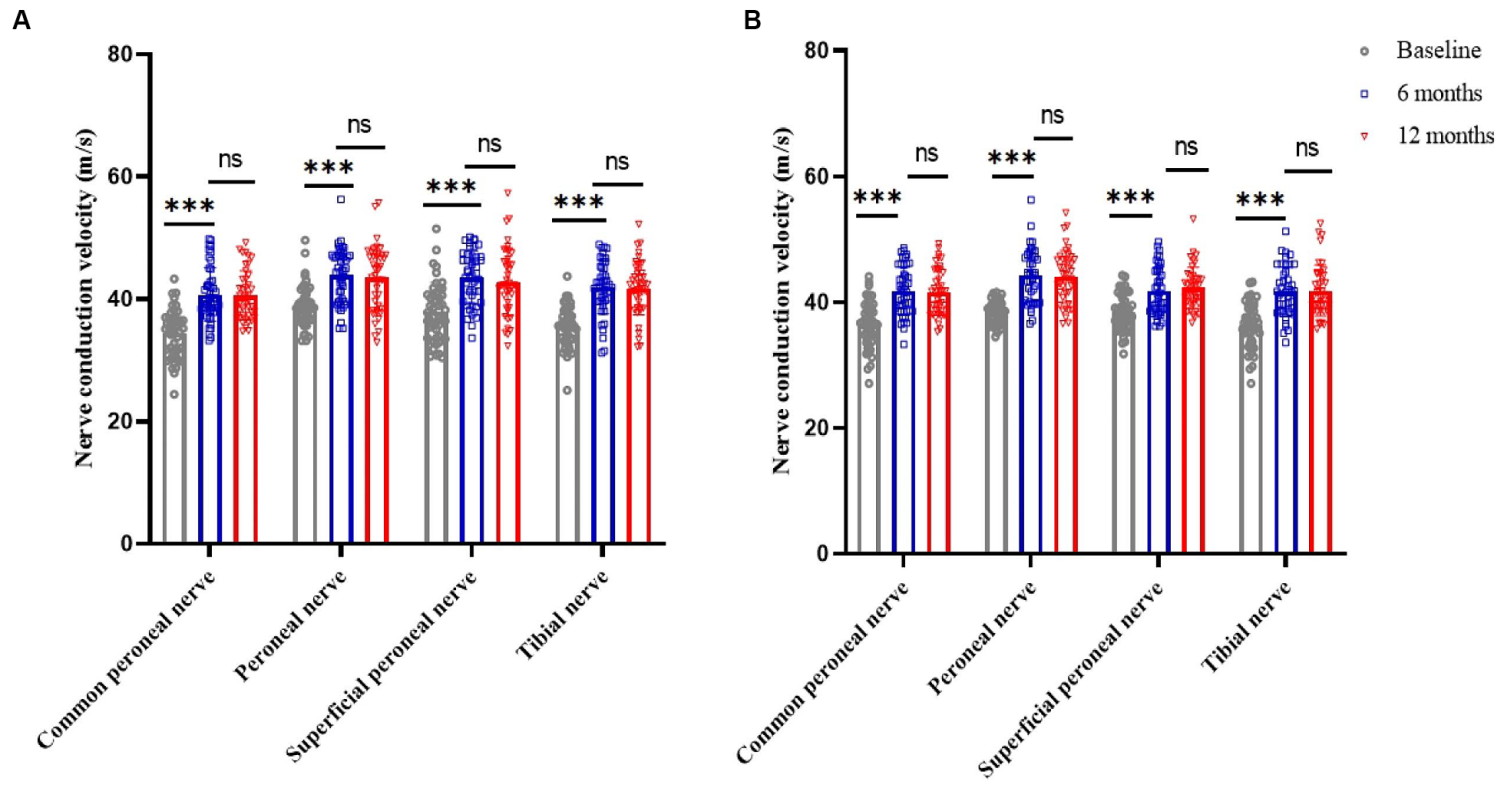
Changes in lower limb nerve conduction velocities at baseline, 6 and 12 months in the SCS and DRG-S groups. ^*^p < 0.05; ^**^p < 0.01; ^***^p < 0.001; NS, *p* > 0.05. **(A)** Lower limb nerve conduction velocity changes in SCS group at baseline, 6 and 12 months; **(B)** Lower limb nerve conduction velocity changes in DRG-S group at baseline, 6 and 12 months.

Compared to baseline, nerve conduction velocities of the common peroneal, peroneal, superficial peroneal and tibial nerves in the lower limbs of patients in the SCS and DRG-S groups were significantly improved at 6 and 12 months postoperatively, respectively, *p* < 0.05. However, there was no significant difference in the changes between 6 and 12 months (Figures 4A,B).

### Glycaemic haemoglobin

At 6 months, HbA1c was 7.86 (0.75) in the SCS group and 7.92 (0.66) in the DRG-S group. At 12 months, HbA1c was 7.90 (0.71) in the SCS group and 7.95 (0.77) in the DRG-S group. There was no significant difference in HbA1c between the two groups at either 6 or 12 months (Table 4).

**Table 4:** Comparison of HbA1c (%) in the SCS and DRG-S groups.

	SCS group		DRG-S group		*p* value
	Mean	SD	Mean	SD	
6-month follow-up	7.86	0.75	7.92	0.66	0.382
12-month follow-up	7.90	0.71	7.95	0.77	0.461

## Discussion

This prospective study showed that patients in both the SCS and DRG-S groups had significantly lower NRS scores at both the 6- and 12-month follow-up compared to baseline. There was no significant difference between the SCS and DRG-S groups at 12 months compared to 6 months. Importantly, the proportion of patients with NRS remission ≥50 in the SCS group was 80.43 and 79.07% at 6 and 12 months, respectively, whereas the proportion of patients with NRS remission ≥50 in the DRG-S group was 79.55 and 80.95%, respectively, and there was no difference between the two groups. In addition, EQ-5D, EQ-VAS and lower limb nerve conduction velocities were significantly improved in all patients who attended follow-up. There was no significant difference in HbA1c between the two groups Thus, we compared that both SCS and DRG-S could improve lower limb nerve conduction function, reduce pain levels and improve quality of life in patients with PDPN.

In a prospective RCT long-term study of SCS, 13 of 22 patients (59%) had ≥50% NRS remission at 6 months; 11 of 17 (65%) had ≥50% NRS remission at 24 months, with 9 (53%) reporting a significant improvement in quality of life; and 7 of 22 (32%) had ≥50% NRS remission at 5 years. 80% of patients with permanent implants were still using their SCS device at 5 years (5–7). A recent RCT showed that when 10 kHz SCS was combined with conventional medical management (CMM), 75 of 95 patients (79%) had >50% pain relief on the Visual Analogue Score (VAS) at 6 months and 121 of 142 patients (85%) had >50% pain relief at 12 months (8–10). In another prospective multicentre RCT comparing DRG-S and SCS in the treatment of complex regional pain syndrome (CRPS), the proportion of DRG-S and SCS groups achieving treatment success (>50% pain relief) at 3 months was 81.2% (56/69) vs. 55.7% (39/70). Percentage of success (74.2%; 49/66 vs. 53.0%; 35/66) (15). These results are consistent with our findings that both SCS and DRG-S are important tools in the treatment of PDPN in terms of neuromodulation and can achieve significant therapeutic results. However, in contrast to other studies, our study did not only compare the improvement in NRS between the two groups as a whole, but also individualised the improvement in each patient (Figure 2). In terms of quality of life, we used the internationally recognised EQ-5D-3L scoring system in our study design. These are the strengths of this study over the existing literature. In addition, our study also showed that the SCS and DPG-S techniques have an effect on HbA1c in diabetic patients.

From an analgesic mechanism perspective, SCS consists of epidural electrodes in the dorsal columns of the spinal cord to excite inhibitory interneurons to release γ-aminobutyric acid (GABA) by retrogradely stimulating Aβ fibres in the spinal cord, which interrupts the transmission of nociceptive signals from the spinal cord to the brain, thereby attenuating the nociceptive signals; dorsal columns of the Aβ fibres can also be positively stimulated to produce paresthesia in the areas innervated by the fibres, masking the painful sensation and thus achieving pain relief (16). With regard to the DRG-S, one study found that there is an extensive GABAergic network between the cell bodies of DRG neurons, and that sensory neurons in the DRG have the ability to express the key proteins required for GABA synthesis and release, and can release GABA when DRG neurons receive a stimulus (22). It has been suggested that DRG-S, like SCS, relies on stimulation of Aβ fibres in the dorsal horn of the spinal cord and release of GABA to activate the pain gating mechanism and inhibit nociception (23, 24). Interestingly, however, a recent study found that the analgesic effect of DRG-S does not depend on the release of GABA in the dorsal horn of the spinal cord (25). Although the analgesic mechanism of DRG-S is highly controversial, it is clear that DRG-S inhibits the excitability of slow pain fibres (C-fibres) (26).

This study has some limitations. First, the process of grouping patients was not randomised, and therefore there is a possibility of selection bias. Fortunately, a comparison of the baseline values of patients in the SCS and DRG-S groups who attended our follow-up showed that the groups were equivalent at baseline and therefore comparable. Second, the follow-up period was only 12 months, the number of cases was small, and only the traditional tonic stimulation mode (voltage, 0.5 V; pulse width, 180–240 μs; frequency, 40 Hz) was used; other stimulation modes or waveforms, such as burst mode and high-frequency stimulation, were not evaluated. Nevertheless, the current results suggest that both SCS and DRG-S are potentially effective treatments for PDPN. In this trial design, we used a single-blind approach to avoid biassing the results, but there are still some potential effects, including rater bias, treatment effect expectation and data collection bias. Although single blinding is not as advantageous as double or triple blinding designs to completely eliminate these potential effects, it is still a commonly used form of blinding that can reduce subjective bias to some extent.

To our knowledge, this is the first cohort study comparing SCS and DRG-S in the treatment of PDPN in clinical research. Most previous SCS studies have focused on the clinical efficacy of lower extremity pain and quality of life in patients with PDPN, and most DRG-S studies have focused on the treatment efficacy of complex regional pain syndrome (CRPS). The design of this study included both SCS and DRG-S groups and assessed the percentage of patients in both cohorts with Numeric Rating Scale (NRS) relief ≥50% at 6 and 12 months, as well as postoperative quality of life scores (EQOL-5D) and effects on HbA1c. These results provide clinicians with higher quality individualised protocols for the treatment of PDPN.

## Data availability statement

The original contributions presented in the study are included in the article/supplementary material; further inquiries can be directed to the corresponding author.

## Ethics statement

The studies involving humans were approved by the Medical Ethics Committee of Shengjing Hospital. The studies were conducted in accordance with the local legislation and institutional requirements. The participants provided their written informed consent to participate in this study.

## Author contributions

Y-FH: Conceptualization, Formal analysis, Investigation, Methodology, Software, Writing – original draft, Writing – review & editing. XC: Data curation, Methodology, Project administration, Supervision, Validation, Visualization, Writing – original draft, Writing – review & editing.
